# Zero-shot multimodal large language models underperform a domain-trained CNN baseline in pediatric wrist fracture detection

**DOI:** 10.1038/s41598-026-58763-w

**Published:** 2026-06-17

**Authors:** Matteo Haupt, David Weiß, Tim Bellersen, Martin H. Maurer

**Affiliations:** Department of Diagnostic and Interventional Radiology, Carl von Ossietzky Universität Oldenburg, Oldenburg, Germany

**Keywords:** Computational biology and bioinformatics, Health care, Medical research

## Abstract

Multimodal large language models (LLMs) that process text and images are increasingly discussed for medical imaging, yet their diagnostic performance on radiographs remains poorly characterized. We evaluated three commercially available multimodal LLMs (GPT-4o, Claude 3.5 Sonnet, Gemini 1.5 Pro) in a strict zero-shot setting for pediatric wrist fracture detection and compared them with a domain-trained Inception v3 convolutional neural network (CNN) on the same GRAZPEDWRI-DX dataset. We constructed a balanced patient-level test cohort of 1,000 children (2,298 radiographs; 500 fracture, 500 non-fracture). The CNN achieved high diagnostic performance (AUROC 0.905, AUPRC 0.920), whereas all LLMs performed close to chance (accuracies < 0.55, Matthews correlation coefficients ≈ 0) and produced bounding boxes often inconsistent with expert annotations. These findings indicate that, in a strict zero-shot setting, the three commercial multimodal LLMs evaluated here lack reliable diagnostic ability for pediatric wrist fracture detection and should therefore be regarded as exploratory research tools rather than clinically dependable systems for pediatric radiograph interpretation.

**Supplementary Information:**

The online version contains supplementary material available at 10.1038/s41598-026-58763-w.

## Introduction

Multimodal large language models (LLMs) capable of processing both text and images have rapidly advanced and are increasingly discussed for clinical applications, including medical image interpretation. Recent models such as GPT-4o, Claude 3.5 Sonnet, and Gemini 1.5 Pro integrate visual and linguistic reasoning and are increasingly explored for medical applications. Early studies show that while these models can summarize radiology reports or answer clinical questions, their consistent diagnostic performance on real radiographs remains limited and insufficiently validated^1,2^. In particular, direct head-to-head comparisons against established supervised baselines on identical clinical test cohorts are currently lacking.

Radiology is a safety-critical diagnostic specialty, where errors may lead to delayed treatment or unnecessary interventions. Tasks such as fracture detection require identifying subtle abnormalities under variable projection quality and anatomical complexity. Missed or delayed diagnosis of pediatric wrist fractures can lead to persistent pain, temporary loss of hand function, and the need for additional follow-up imaging or treatment, underscoring the clinical importance of reliable detection^3^. Pediatric musculoskeletal radiographs are particularly challenging: growth plates, secondary ossification centers, and overlapping structures often mimic or obscure cortical disruptions^4,5^. These factors make pediatric distal forearm fractures a stringent benchmark for evaluating the visual-reasoning capabilities of general-purpose multimodal models.

Despite their rapid adoption and widespread availability through commercial APIs, multimodal LLMs remain largely untested for direct image-based diagnostic tasks on radiographic datasets. Most existing evaluations focus on natural-image benchmarks, text-generation tasks, or radiology report summarization, rather than direct classification or localization of pathology^6,7^. Initial diagnostic comparisons of multiple commercial multimodal LLMs have appeared on text-rich radiology cases^8^, and pediatric musculoskeletal applications have been preliminarily explored on isolated tasks^9^, but a systematic head-to-head benchmark against domain-trained supervised models on identical pediatric radiograph cohorts is currently lacking. Whether such models achieve reliable diagnostic performance on clinical imaging in a strict zero-shot setting against domain-trained supervised baselines remains insufficiently characterized. Their ability to localize pathology is even less well characterized: although current APIs can output bounding-box coordinates, the spatial accuracy of these predictions has not been rigorously evaluated against expert annotations.

Supervised convolutional neural networks (CNNs) remain a well-established standard for medical image analysis and have demonstrated strong performance on fracture detection tasks, including pediatric wrist trauma^5,10,11^. Providing direct comparative evaluations between such conventional supervised models and modern multimodal LLMs is important to contextualize the capabilities and current limitations of general-purpose LLMs within established diagnostic workflows.

The pre-specified question of this study was not whether zero-shot multimodal LLMs achieve performance comparable to a supervised baseline, but how large the performance gap is on identical pediatric radiographs, and what specific failure modes the LLMs exhibit. In this study, we systematically evaluate the zero-shot diagnostic performance of three contemporary multimodal LLMs—GPT-4o, Claude 3.5 Sonnet, and Gemini 1.5 Pro—for pediatric wrist fracture detection using the public GRAZPEDWRI-DX dataset. We directly compare their patient-level accuracy, error patterns, and localization behavior with a domain-trained CNN baseline on the same dataset. Specifically, we (i) construct a balanced patient-level benchmark cohort of pediatric wrist radiographs, (ii) perform a head-to-head comparison between a weights-accessible, domain-trained CNN and three zero-shot multimodal LLMs on identical clinical images, and (iii) jointly analyze classification performance and exploratory localization behavior based on model-generated bounding boxes, including quantitative IoU-based assessment. To our knowledge, this is the first head-to-head benchmark directly comparing zero-shot multimodal LLMs with a supervised CNN on a clinical radiograph dataset of pediatric wrist fractures. This study aims to provide a rigorous benchmark of current multimodal LLM capabilities for radiographic detection and localization of pediatric distal forearm and wrist fractures, and to highlight key limitations that must be addressed before such systems can be considered for clinical deployment.

## Results

**Fig. 1:**
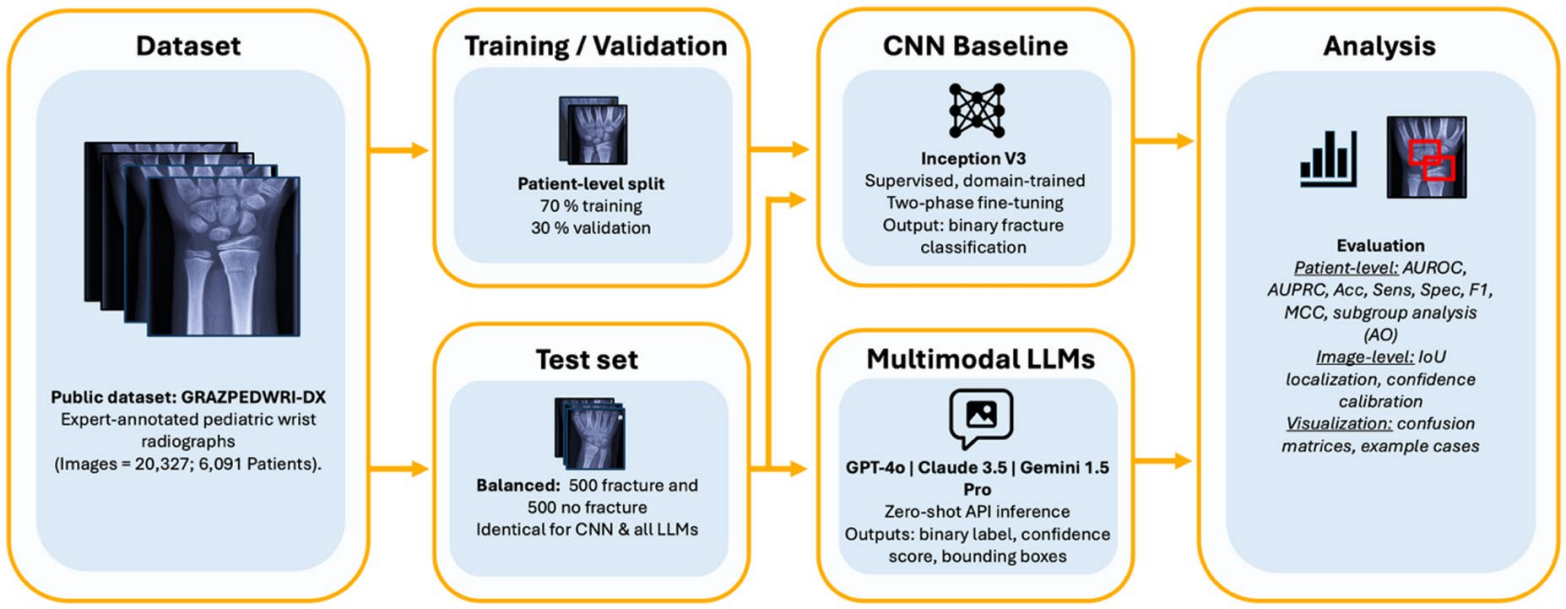
Experimental pipeline comparing zero-shot multimodal large language models (LLMs) with a convolutional neural network (CNN) for pediatric wrist fracture detection. Radiographs from the public GRAZPEDWRI-DX dataset (20,327 images, 6,091 patients) were split on the patient level for CNN training and a balanced test set (500 fracture, 500 non-fracture) used identically for all models. The CNN baseline (Inception v3) was trained in two supervised phases, while GPT-4o, Claude 3.5 Sonnet, and Gemini 1.5 Pro were evaluated in a zero-shot multimodal setting. Evaluation included patient-level classification metrics (accuracy, sensitivity, specificity, F1, MCC; AUROC and AUPRC for the CNN), subgroup analysis by AO fracture subtype, quantitative bounding-box localization (IoU), and analysis of LLM confidence-score calibration. Abbreviations: AUPRC = area under the precision-recall curve; AUROC = area under the receiver-operating-characteristic curve; CNN = convolutional neural network; F1 = F1-score; IoU = intersection-over-union; LLM = large language model; MCC = Matthews correlation coefficient.

An overview of the study design, dataset construction, and evaluation workflow is shown in Fig. 1.

### Test cohort characteristics

The balanced patient-level test cohort comprised 1,000 pediatric patients with 2,298 wrist radiographs (500 fracture, 500 non-fracture). The mean age was 10.8 years (SD 3.9; range 0.6–18.9), and 52% of patients were male. Almost all patients had both anteroposterior and lateral projections (96.8%). The specific cohort characteristics are summarized in Supplementary Table S1. All models were evaluated on this identical test cohort to ensure direct comparability.

### Diagnostic performance of the CNN baseline

**Fig. 2:**
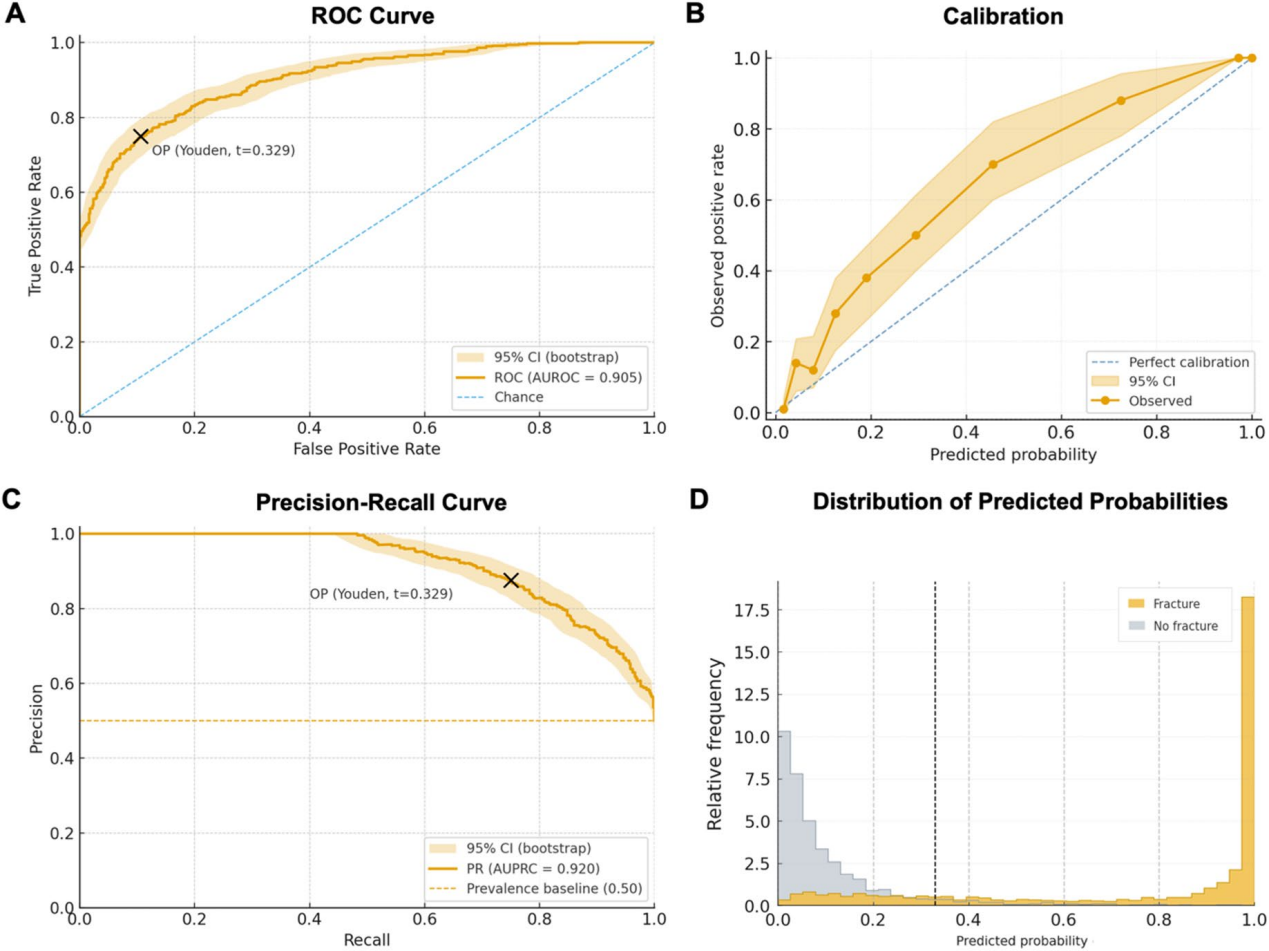
Performance of the convolutional neural network (CNN) baseline on the patient-level test set. **(A)** ROC curve with AUROC = 0.905 (95% CI 0.887–0.923). The optimal operating point (Youden’s J = 0.329) is indicated. **(B)** Calibration curve showing observed versus predicted positives (95% bootstrap CIs). **(C)** Precision–recall (PR) curve with AUPRC = 0.920 (95% CI 0.903–0.936) and the prevalence baseline (0.50). **(D)** Distribution of CNN-predicted probabilities for fracture (orange) and non-fracture (grey); the dashed line indicates the Youden’s J threshold.

The convolutional neural network achieved a balanced accuracy of 0.821 on the patient-level test set. At the operating point determined by Youden’s J (t = 0.329), the CNN reached a sensitivity of 0.75 (95% CI 0.71–0.79) and a specificity of 0.89 (95% CI 0.87–0.92). Overall accuracy was 0.82 (95% CI 0.80–0.85), with a Matthews correlation coefficient (MCC) of 0.65 (95% CI 0.60–0.70) (Table 1). The receiver-operating-characteristic curve yielded AUROC = 0.905 (95% CI 0.887–0.923), and the precision–recall curve showed AUPRC = 0.920 (95% CI 0.903–0.936) (Fig. 2A and C). These results reflect strong concordance between model predictions and expert annotations. The CNN also demonstrated broadly consistent calibration between predicted risk and observed fracture rates (Fig. 2B). The distribution of predicted fracture probabilities showed a clear separation between fracture and non-fracture cases, with most fracture predictions concentrated above the Youden-optimal threshold (Fig. 2D). The CNN was also retrained with four additional random seeds (123, 777, 999, 2024). Performance was consistent across runs (Supplementary Table S3).

**Table 1:** Patient-level diagnostic performance of the CNN baseline and multimodal LLMs on the balanced pediatric wrist radiograph test set. Values show 95% bootstrap confidence intervals (2,000 resamples). Balanced accuracy (BA) was estimated by the same patient-level resampling. AUROC and AUPRC were reported for the CNN only.

Model	Sensitivity (95% CI)	Specificity (95% CI)	PPV (95% CI)	NPV (95% CI)	Accuracy (95% CI)	F1-score (95% CI)	MCC (95% CI)	AUROC (95% CI)	AUPRC (95% CI)	Balanced Accuracy (95% CI)
CNN Baseline (Inception v3)	0.748 (0.709–0.786)	0.894 (0.866–0.921)	0.876 (0.844–0.908)	0.780 (0.746–0.815)	0.821 (0.797–0.845)	0.807 (0.778–0.835)	0.649 (0.603–0.696)	0.905 (0.887–0.923)	0.920 (0.903–0.936)	0.821 (0.797–0.845)
GPT-4o	0.854 (0.823–0.884)	0.144 (0.114–0.176)	0.499 (0.466–0.533)	0.497 (0.418–0.582)	0.499 (0.469–0.530)	0.630 (0.600–0.659)	–0.003 (–0.064–0.063)	–	–	0.499 (0.478–0.522)
Claude 3.5 Sonnet	0.290 (0.252–0.330)	0.700 (0.659–0.741)	0.492 (0.434–0.548)	0.496 (0.461–0.534)	0.495 (0.465–0.527)	0.365 (0.323–0.408)	–0.011 (–0.072–0.050)	–	–	0.495 (0.487–0.512)
Gemini 1.5 Pro	0.402 (0.360–0.444)	0.690 (0.649–0.731)	0.565 (0.511–0.615)	0.536 (0.498–0.573)	0.546 (0.517–0.576)	0.470 (0.428–0.509)	0.096 (0.035–0.155)	–	–	0.546 (0.519–0.575)

### Zero-shot performance of multimodal large language models

**Fig. 3:**
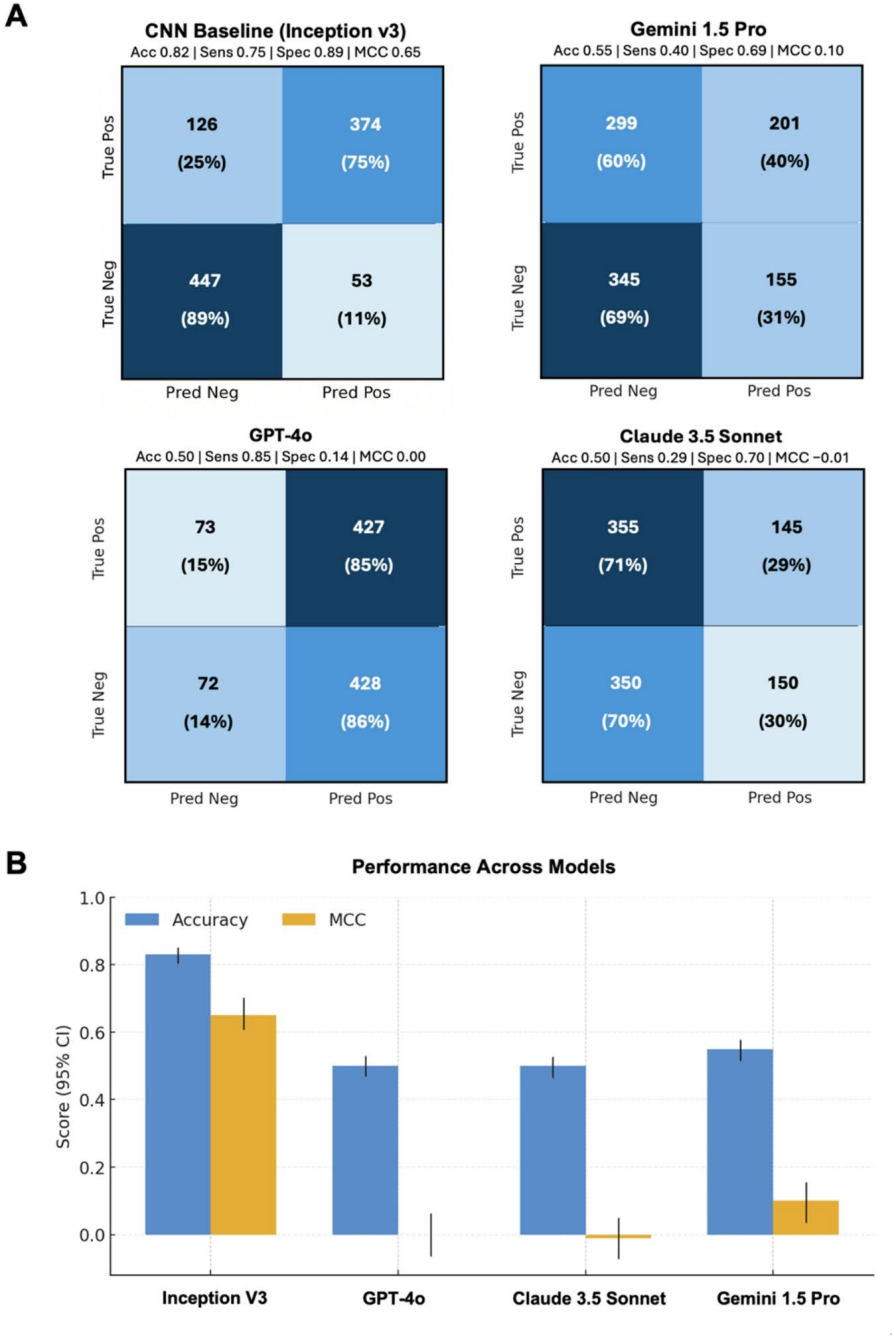
Comparative diagnostic performance of the CNN baseline and multimodal large language models (LLMs). **(A)** Patient-level confusion matrices for the CNN baseline (Inception v3) and three LLMs (GPT-4o, Claude 3.5 Sonnet, Gemini 1.5 Pro). Rows correspond to the ground-truth class (True Pos = fracture, True Neg = no fracture) and columns to the predicted class (Pred Neg, Pred Pos): false negatives (upper left), true positives (upper right), true negatives (lower left), and false positives (lower right) are shown as counts, with row-wise percentages in parentheses. Header lines report accuracy (Acc), sensitivity (Sens), specificity (Spec), and Matthews correlation coefficient (MCC). The CNN achieved robust sensitivity (0.75) and high specificity (0.89) with high overall accuracy (0.82), while all LLMs performed near chance. **(B)** Accuracy (blue) and MCC (orange) with 95% confidence intervals derived from 2,000 patient-level bootstrap resamples. The CNN markedly outperformed all LLMs, whose MCC values approached zero; non-overlapping bootstrap confidence intervals indicate robust performance differences.

All three multimodal LLMs performed close to chance under zero-shot conditions (Table 1). GPT-4o adopted a strongly sensitivity-biased operating pattern, detecting most fractures (sensitivity 0.854, 95% CI 0.823–0.884) but at the cost of extremely low specificity (0.144, 95% CI 0.114–0.176). As a result, it correctly identified 427 of 500 fracture patients (TP) and 73 fracture patients were missed (FN), while only 72 of 500 non-fracture patients were correctly classified (TN) and 428 were falsely flagged (FP; Fig. 3A).

In contrast, Claude 3.5 Sonnet and Gemini 1.5 Pro operated in a specificity-favoring regime, with specificities of 0.700 (95% CI 0.659–0.741) and 0.690 (95% CI 0.649–0.731), respectively, but very low sensitivities (0.290 and 0.402; Table 1), indicating systematic under-detection of fractures. Patient-level counts were 145 TP / 355 FN / 350 TN / 150 FP for Claude 3.5 Sonnet and 201 TP / 299 FN / 345 TN / 155 FP for Gemini 1.5 Pro (Fig. 3A). Across all LLMs, overall accuracies remained ≤ 0.546 and balanced accuracies approximated 0.50, while MCC values ranged from − 0.011 to 0.096; for GPT-4o and Claude 3.5 Sonnet, the 95% CIs crossed zero, and for Gemini 1.5 Pro the MCC remained only slightly above zero, consistent with near-random discriminative performance. Bootstrap confidence intervals for accuracy and MCC did not overlap with those of the CNN baseline (Fig. 3B), indicating large and robust descriptive performance differences.

### Performance across fracture subtypes

In an exploratory subgroup analysis (Supplementary Table S4), the CNN reached its highest sensitivity on displaced/complete fractures (AO /3, 0.956, 95% CI 0.889–1.000) and Salter–Harris injuries (AO /7, 0.854, 95% CI 0.732–0.951), with somewhat lower performance on the predominant subtype of subtle buckle/torus injuries (AO /2, 0.717, 95% CI 0.672–0.763), which constituted 79% of the fracture cohort. The three LLMs showed a markedly flatter profile across strata: GPT-4o remained close to its overall sensitivity (~ 0.85) regardless of fracture subtype, while Claude 3.5 Sonnet stayed below 0.40 in every subgroup—including displaced fractures, where it missed three quarters of cases (0.244, 95% CI 0.133–0.378). Gemini 1.5 Pro showed only a small relative increase on displaced fractures (0.556, 95% CI 0.422–0.689) and on cases with ulna involvement (0.532, 95% CI 0.403–0.661).

### Localization and bounding-box performance

**Fig. 4:**
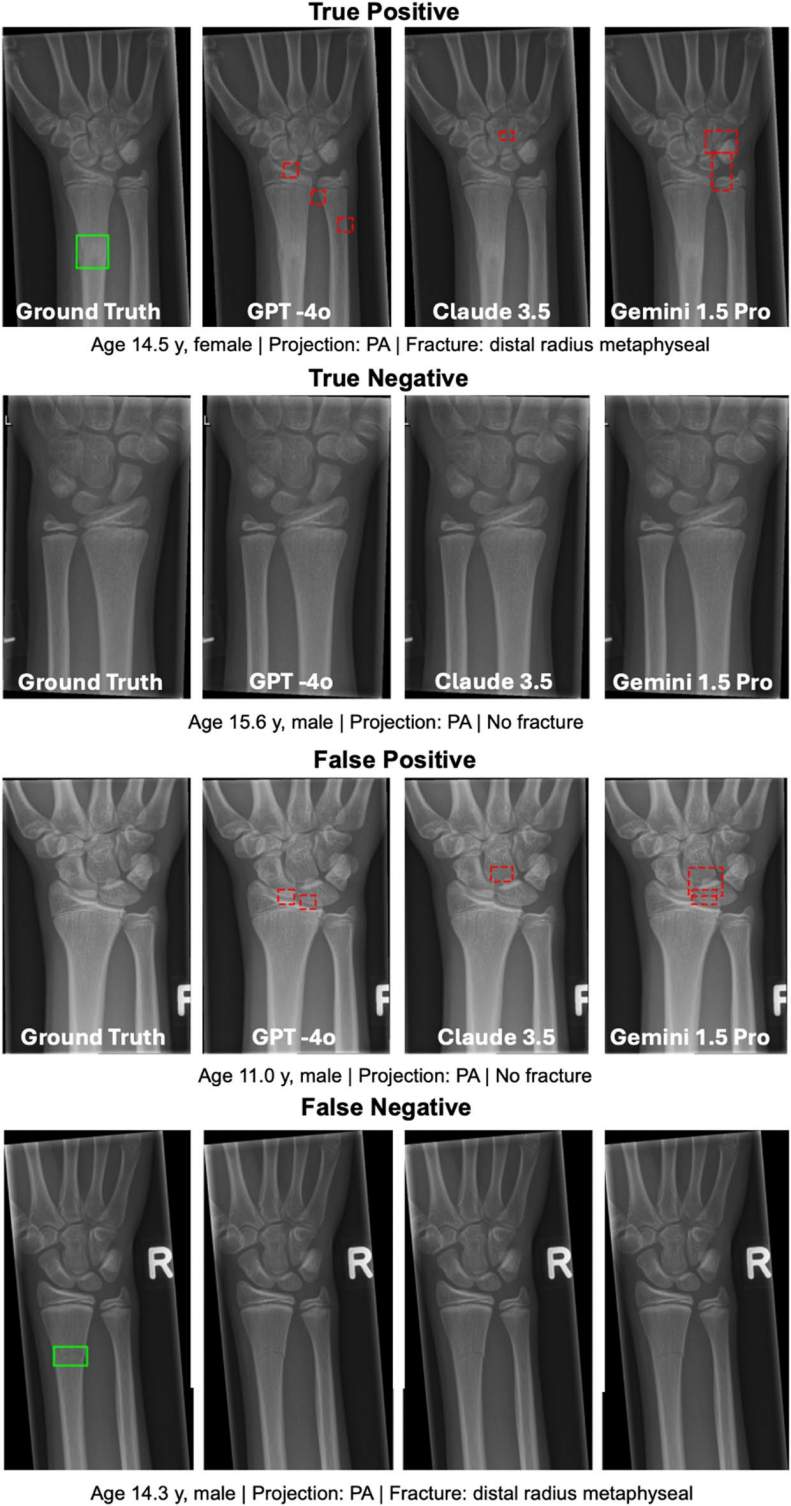
Representative examples of multimodal large language model (LLM) predictions for pediatric wrist fractures. Shown are four illustrative cases demonstrating qualitative model behavior across true positive, true negative, false positive, and false negative classifications. For each case, the radiological ground truth is displayed in the leftmost column, followed by the corresponding predictions from GPT-4o, Claude 3.5 Sonnet, and Gemini 1.5 Pro. Green boxes indicate the annotated fracture location in the ground-truth reference images; red boxes mark the model-predicted locations when a fracture was identified. Each row includes patient age and projection to provide clinical context.

Bounding-box predictions produced by all LLMs showed minimal spatial agreement with expert annotations. Although the models often returned visually plausible boxes, qualitative review revealed that these rarely corresponded to the true fracture sites (Fig. 4). Typical failure modes included boxes placed on open growth plates, metaphyseal regions without cortical disruption, or soft tissue, as well as complete omission of clearly visible fractures. Quantitative localization analysis on fracture-positive images (*n* = 1,009) confirmed this pattern (Supplementary Table S6). Mean IoU between predicted and ground-truth boxes was below 0.1 for all three models (GPT-4o 0.072, Claude 0.022, Gemini 0.094). The fraction of fracture-positive images for which a model produced a box overlapping the ground truth at the standard IoU ≥ 0.5 threshold was 0.0–0.6%, and remained at most 2.4% even at the lenient IoU ≥ 0.3 threshold. Localization findings are therefore reported as exploratory.

### Confidence calibration of LLM predictions

Although each prompt requested a numeric confidence score (0–100), and each radiograph was independently scored, none of the LLMs provided a useful self-assessment signal at the image level (*n* = 2,298 image-level predictions; Supplementary Table S5). Gemini 1.5 Pro returned a confidence of 90 for 99.4% of images (range 90–95), effectively ignoring the calibration rubric. GPT-4o used the score primarily as a binary alias of its label, assigning high confidence to most “yes” predictions and low confidence to most “no” predictions. Claude 3.5 Sonnet operated in a narrow high-confidence range (median 95 for “no” predictions, 90 for “yes” predictions). When the signed confidence (confidence/100 if “yes”, 1 − confidence/100 if “no”) was used directly as a continuous decision score, image-level AUROC remained essentially at chance level for all three models (GPT-4o 0.493, 95% CI 0.482–0.505; Claude 0.488, 95% CI 0.465–0.511; Gemini 0.518, 95% CI 0.503–0.534), and mean confidence on correct versus incorrect image-level predictions did not differ meaningfully (GPT-4o 56.1 vs. 65.4; Claude 79.0 vs. 79.2; Gemini 90.0 vs. 90.0). The reported confidence scores therefore did not carry diagnostic information beyond the binary label, which is why all headline metrics were based on the label.

### Direct comparison between supervised and zero-shot models

Across all diagnostic metrics, the supervised CNN substantially outperformed every evaluated LLM. The CNN maintained robust sensitivity, high specificity, and overall diagnostic accuracy, whereas all LLMs demonstrated near-random classification behavior and qualitatively poor localization. Absolute accuracy differences between the CNN and each LLM were large, ranging from approximately + 28 to + 33% points. Together, these results show that a simple, domain-trained CNN exhibits strong and stable diagnostic performance, while the three zero-shot multimodal LLMs evaluated here (GPT-4o, Claude 3.5 Sonnet, Gemini 1.5 Pro) lack the ability to detect pediatric wrist fractures reliably under these conditions.

## Discussion

This study provides a systematic evaluation of three contemporary multimodal large language models - GPT-4o, Claude 3.5 Sonnet, and Gemini 1.5 Pro - for pediatric wrist fracture detection in a strict zero-shot setting. Across a balanced cohort of 1,000 pediatric patients, all LLMs demonstrated diagnostic performance close to chance, with accuracies below 0.55 and Matthews correlation coefficients near zero. In contrast, an Inception v3 convolutional neural network trained on the same dataset achieved high and reasonably calibrated performance with an AUROC of 0.905, AUPRC of 0.920, and a robust sensitivity–specificity balance. These findings indicate that, despite recent progress in multimodal reasoning, current general-purpose LLMs do not reliably detect subtle radiographic abnormalities without domain-specific adaptation.

Our findings are consistent with reports that foundation models, despite strong natural-image performance, have not yet demonstrated robust generalization to clinical imaging tasks requiring fine-grained spatial detail^12–14^. Although several studies have highlighted the potential of LLMs for radiology report summarization, clinical question answering, or workflow support, direct diagnostic evaluations remain limited. Recent systematic reviews have shown that general-purpose LLMs can generate clinically unreliable or incorrect recommendations in medical scenarios^15,16^. Similar concerns have emerged in cardiology and other specialties, where interactive LLM-based tools sometimes provide inappropriate or incomplete suggestions^17^. These observations are consistent with analyses highlighting substantial limitations of foundation models in safety-critical clinical applications^18^.

Pediatric wrist fractures constitute a particularly challenging benchmark for general-purpose systems. Growth plates, secondary ossification centers, and subtle cortical irregularities frequently mimic or obscure true fractures, making them difficult even for trained radiologists and diagnostic errors occur frequently^4,19,20^. The consistently poor zero-shot performance across three independent LLM architectures suggests that the general-purpose visual representations available through these closed models do not reliably capture the domain-specific radiographic cues required for pediatric wrist fracture detection. Beyond classification accuracy, the LLMs also failed to provide useful self-assessment: their reported confidence scores either tracked the binary label (GPT-4o), saturated near the upper bound regardless of correctness (Claude), or ignored the rubric entirely (Gemini, returning 90 for 99.4% of cases). This absence of meaningful uncertainty information further limits the suitability of current general-purpose LLMs as clinical decision support.

The exploratory subgroup analysis suggested severity-dependent behavior of the supervised CNN, with higher sensitivity for displaced/complete fractures and Salter–Harris injuries than for subtle buckle/torus fractures, which represented the predominant fracture subtype in the present cohort. This finding is consistent with prior work showing that test-set composition and fracture subtype distribution can substantially influence AI performance in pediatric wrist fracture detection^21^. In contrast, the LLMs showed comparatively flat sensitivity profiles across fracture subtypes, indicating limited responsiveness to radiographic fracture severity. Because this exploratory analysis was restricted to fracture-positive patients and therefore did not capture subtype-specific false-positive behavior, it should not be interpreted as a complete subtype-specific diagnostic performance comparison.

Quantitative localization metrics confirmed this pattern, with mean IoU below 0.1 for all three models and IoU ≥ 0.5 coverage of at most 0.6% across the 1,009 fracture-positive images. These results extend prior reports of near-zero spatial agreement of vision–language models on medical images to the pediatric musculoskeletal setting^12^.

Several limitations of this study warrant consideration. The analysis was restricted to a single dataset and anatomical region; external validation across institutions and imaging modalities is required to assess generalizability. In addition, the test cohort was intentionally balanced (50% fractures, 50% non-fractures) to facilitate model comparison, so positive and negative predictive values do not reflect real-world fracture prevalence in emergency departments and should not be directly extrapolated to clinical practice. All LLMs were evaluated exclusively in a strict zero-shot setting via closed-source APIs, which limits insight into pretraining composition or internal mechanics. We did not perform task-specific calibration, threshold tuning, or few-shot prompting; instead, we used a single standardized instruction prompt for all models. Although this reflects typical “plug-and-play” usage conditions for general-purpose LLMs in clinical environments, it prevents exploration of fine-tuned or radiology-adapted variants that may perform better, even though such configurations are not yet widely accessible for clinical radiographs. More fundamentally, the three multimodal LLMs evaluated here are accessible only through commercial APIs, with no public weights and only limited fine-tuning interfaces, so large-scale image-conditioned adaptation of these specific models on radiographic data is not currently feasible for external researchers^22^. The fine-tuning asymmetry between a domain-trained CNN and zero-shot closed-API LLMs is therefore a structural feature of comparison studies of this kind, and conclusions about the LLMs apply to their as-deployed behavior rather than to a hypothetical radiology-adapted variant. Open-weights multimodal models and broader image-fine-tuning APIs are expected to enable more symmetric comparisons in future work. LLM outputs are also known to be sensitive to prompt wording, and visual artifacts on radiographs can act as incidental prompt injections that distort vision–language model responses^23^. Conclusions drawn from any single prompting strategy are therefore necessarily limited. To minimize artifact-related confounding, our test cohort was deliberately restricted to images free of casts, metallic implants, and diagnostic-uncertainty markers. While these constraints reflect the pre-specified scope of a strict zero-shot, plug-and-play benchmark, a few-shot arm with curated in-context exemplars (e.g., 5–10 radiographs) would help disentangle which observed failure modes are inherent to current multimodal LLMs and which are artifacts of zero-shot prompting^24^. Several of the present findings are, however, unlikely to be primarily zero-shot artifacts: the near-zero localization IoU (Supplementary Table S6) reflects a structural limitation of current vision encoders rather than a missing prompt context, and the rubric-blind confidence-score behavior (Supplementary Table S5) is independent of label content. LLM inference is also known to exhibit session-level variability, introducing potential stochasticity. The CNN baseline, although intentionally relatively simple and easy to reproduce, is not intended to represent the upper bound of supervised performance. Modern supervised approaches in medical imaging often achieve substantially higher accuracy through deeper architectures, model ensembles, or transformer-based designs^25,26^. While a transformer-based supervised baseline such as the Vision Transformer would offer a more architecturally symmetric comparison with the Transformer-based multimodal LLMs evaluated here, vision transformers typically require substantially larger training datasets to outperform CNN baselines on tasks of the present scale^27^. However, the aim of this study was not to establish a new state-of-the-art CNN, but to use a well-characterized baseline for direct comparison with zero-shot multimodal LLMs. Despite these limitations, the central conclusion is clear: in a strict zero-shot setting, the three commercial multimodal LLMs evaluated here (GPT-4o, Claude 3.5 Sonnet, and Gemini 1.5 Pro) lack reliable diagnostic ability for pediatric wrist fracture detection and remain substantially inferior to a supervised CNN baseline trained on a modest domain-specific dataset. Clinical deployment without rigorous validation risks harmful error patterns, including missed fractures and excessive false positives. While multimodal LLMs may hold value for educational applications, structured report generation, or non-diagnostic triage support, safe integration into radiology workflows will require radiology-specific fine-tuning, transparent evaluation frameworks, and continuous model auditing. Continued development of domain-adapted multimodal architectures and open benchmarking resources will be crucial to bridge the gap between general foundation models and clinically dependable diagnostic tools. For digital health stakeholders and regulators, our findings underscore the need for dedicated evaluation frameworks that explicitly test foundation models on high-stakes imaging tasks before they are considered as medical devices or integrated into clinical decision support. Benchmarks such as this one may help delineate safe use cases, inform risk mitigation strategies, and guide the development of radiology-specific multimodal architectures.

## Methods

### Dataset and cohort

This study used the publicly available GRAZPEDWRI-DX dataset, which contains 20,327 pediatric wrist radiographs from 6,091 patients collected at the University Hospital Graz between 2008 and 2018^28^. Each image was annotated by expert radiologists for fracture presence and localization. For evaluation, a balanced patient-level test cohort of 1,000 patients (500 fracture, 500 non-fracture) was defined, comprising 2,298 radiographs (1,009 fracture-positive and 1,289 non-fracture at the image level). Patient inclusion required all of the patient’s images to be free of casts, metallic implants, or diagnostic-uncertainty markers in the source dataset, ensuring that the test cohort was unaffected by potential visual artifacts from clinical hardware or ambiguous expert annotations. This identical test set was used for both the convolutional neural network baseline and the large language model evaluations to ensure direct comparability. The remaining patients (*N* = 5,091) served exclusively for CNN training and validation, with patient-wise splits to avoid subject overlap between training, validation, and test sets. Patients in this non-test subset were randomly partitioned at the patient level into a training set (70%) and a validation set (30%).

### Multimodal large language model inference

Three commercially available multimodal LLMs were tested in a zero-shot configuration: GPT-4o (OpenAI), Claude 3.5 Sonnet (Anthropic), and Gemini 1.5 Pro (Google DeepMind). In this study, zero-shot evaluation denotes inference without any task-specific fine-tuning, gradient updates, or inclusion of labeled examples in the prompt; a single standardized instruction prompt was used for all models, following common usage in large language model evaluation^29^. The pretraining data of closed-source models are undisclosed; we make no claims about potential prior exposure to radiographs. Each model was accessed via its official API during August–September 2025, using the default endpoints for the gpt-4o (OpenAI), claude-3-5-sonnet (Anthropic), and gemini-1.5-pro (Google DeepMind) model families, which served the snapshots gpt-4o-2024-11-20, claude-3-5-sonnet-20,241,022, and gemini-1.5-pro-002. All calls used identical prompting with a temperature of 0 and enforced JSON output. Radiographs were transmitted individually in their original PNG format and native resolution as provided by GRAZPEDWRI-DX, without any additional preprocessing, rescaling, or compression. A standardized system-and-user-prompt pair defined a research context and requested structured JSON output containing a binary fracture label and up to three bounding boxes. Each prompt explicitly requested a numeric confidence score (0–100) for the binary prediction. The exact prompt wording used for all LLMs is listed in Supplementary Table S2. All primary performance metrics are based on the binary “label” output; the rationale, including an exploratory analysis of the LLM confidence scores, is provided in the Results. Outputs were parsed automatically, validated for format compliance, and stored for image- and patient-level analysis. At the patient level, results were aggregated with a logical OR rule: if any view was classified positive, the patient was considered positive.

### CNN baseline

As a supervised benchmark, a convolutional neural network using the Inception v3 architecture was trained using TensorFlow 2.15 and Keras 3 on macOS (Apple M-series)^30^. Images were resized to 299 × 299 pixels, normalized to [–1, 1], and augmented with horizontal flips and minor brightness/contrast variation. We optimized a binary cross-entropy loss with mini-batches of 32 images for up to 20 epochs (8 epochs with the convolutional base frozen and up to 12 fine-tuning epochs), using class weights derived from training-set label frequencies, early stopping on validation AUROC (patience 5), and learning-rate reduction on plateau. Training followed a two-stage fine-tuning procedure in which the ImageNet-pretrained convolutional base was initially frozen while a new dense classification head was trained, after which the upper 60 convolutional layers were unfrozen and fine-tuned using Adam optimizers with learning rates of 10⁻³ in the first training stage and 10⁻⁴ in the second. Patient-wise splits prevented subject overlap between training, validation, and test sets. The model was optimized on the training set and validated using AUROC; Youden’s J defined the operating point^31^. To assess robustness, the full pipeline was repeated with four additional random seeds (123, 777, 999, 2024) besides the primary run with seed 42. For comparability, all models were evaluated using the same Youden’s J threshold (t = 0.329) derived from the validation set of the primary run (seed 42). Performance metrics were computed on the identical balanced patient-level test set (*n* = 1,000). At the patient level, we applied the same logical OR aggregation across available views (thresholded per-view predictions) as for the LLMs.

### Evaluation and statistics

Model performance was evaluated at the patient level. Primary metrics included accuracy, sensitivity, specificity, F1-score, Matthews correlation coefficient (MCC), and - for the CNN only - AUROC and AUPRC. Balanced accuracy (BA), defined as the mean of sensitivity and specificity, was considered the primary endpoint. AUROC and AUPRC were computed for the CNN only. Calibration of the CNN baseline was assessed using a reliability curve by grouping predicted probabilities into deciles and plotting the observed fracture rate with 95% bootstrap confidence intervals. All 95% confidence intervals were computed by non-parametric bootstrap with 2,000 resamples and the percentile method^32^. Patients were the unit of resampling throughout. For headline patient-level metrics, each patient counted as one observation (image-level predictions had been aggregated by the logical-OR rule). For image-level exploratory analyses involving multiple correlated radiographs per patient (Supplementary Tables S5 and S6), all images of a resampled patient were retained together (cluster bootstrap), preserving within-patient correlation. Results therefore reflect per-patient diagnostic performance on the balanced cohort (*N* = 1,000). Comparative interpretations are descriptive and based on overlap of bootstrap intervals; no formal significance testing was performed.

For an exploratory subgroup analysis (Supplementary Table S4), fracture-positive patients were stratified by AO severity (maximum severity code across their fracture images) and bone involvement (radius isolated vs. any ulna). Ten patients without a parseable AO severity code were excluded from severity subgroups. Bone-involvement subgroups additionally excluded patients whose AO codes did not specify the affected bone (e.g., ‘23-M/2.1’ without an explicit ‘r’ or ‘u’ prefix), leaving 424 of 500 fracture-positive patients eligible for the radius-versus-ulna stratification. For exploratory localization analysis on fracture-positive images, predicted bounding boxes were compared to expert ground-truth annotations using Intersection-over-Union (IoU). When multiple boxes were predicted or annotated, the best-matching IoU across all predicted-to-ground-truth pairs was retained per image. Hit rates were computed at IoU thresholds of 0.5 and 0.3. The image-diagonal-normalized center-to-center distance was used as a complementary metric. Cases in which the model returned a positive label without an accompanying bounding box would have been counted as misses, but did not occur in the dataset. All three LLMs returned at least one box for every ‘yes’ prediction.

## Supplementary Information

Below is the link to the electronic supplementary material.

Supplementary Material 1

## Data Availability

All data analyzed in this study originate from the publicly available GRAZPEDWRI-DX dataset which contains pediatric wrist radiographs with expert annotations. The dataset can be accessed through the Figshare repository at https://figshare.com/articles/dataset/GRAZPEDWRI-DX/14825193. Processed evaluation files supporting the findings of this study are available from the corresponding author upon reasonable request.

## Code availability

The code used for CNN training and large language model evaluation is available from the corresponding author upon reasonable request.
