# Supplementary material for: Zero-shot multimodal large language models underperform a domain-trained CNN baseline in pediatric wrist fracture detection
Source: Sci Rep. 2026 Jun 17;16:18873. doi: 10.1038/s41598-026-58763-w (PMC13276184; doi:10.1038/s41598-026-58763-w)
Supplement: Supplementary file 1 — Supplementary Material 1 [file 41598_2026_58763_MOESM1_ESM.docx]

**Supplements**

**Supplementary Table S1.** Cohort characteristics of the balanced pediatric wrist radiograph test set (N = 1,000 patients; 500 with fractures, 500 without). The table summarizes age, gender, and radiographic projection coverage. Almost all patients had both AP and LAT projections available, ensuring that the test set reflected routine clinical imaging conditions.

| Variable | Total (N patients) | Fracture visible (n, %) | No fracture (n, %) |
| --- | --- | --- | --- |
| N patients | 1,000 | 500 | 500 |
| Age (years) | 10.84 ± 3.91 (range 0.6–18.9) | 9.92 ± 3.79 (range 0.8–18.9) | 11.76 ± 3.82 (range 0.6–18.0) |
| Gender \| Male | 522 (52.2%) | 286 (57.2%) | 236 (47.2%) |
| Gender \| Female | 478 (47.8%) | 214 (42.8%) | 264 (52.8%) |
| Projection \| AP present | 979 (97.9%) | 496 (99.2%) | 483 (96.6%) |
| Projection \| Lateral present | 989 (98.9%) | 494 (98.8%) | 495 (99.0%) |
| Projection (exclusive) \| Both | 968 (96.8%) | 490 (98.0%) | 478 (95.6%) |

**Supplementary Table S2. Standardized prompt structure used for all multimodal large language models (LLMs).**

| Prompt component | Exact text (identical for GPT-4o, Claude 3.5 Sonnet, and Gemini 1.5 Pro) | Purpose |
| --- | --- | --- |
| **System prompt** | "You are an experienced pediatric radiologist working in a research and education setting. You DO NOT provide medical advice or diagnosis. Goal: decide if a likely fracture is present (binary) on the provided image. If present, return up to THREE representative tight bounding boxes. Always return STRICT JSON only." | Defines a controlled, non-diagnostic research context. Instructs the model to provide structured JSON output only. |
| **User prompt** | "Analyze the pediatric wrist X-ray non-diagnostically. Coordinates: pixel, origin=(0,0) top-left; x→right, y→down; image_size=({W}x{H}). Calibration rubric for 'confidence' (integer 0–100): Very strong evidence: 90–95; Strong evidence: 75–89; Moderate: 55–74; Weak/Equivocal: 35–54; No convincing evidence: 10–34. Avoid defaulting to 85 or 90; choose the narrowest bin matching the evidence. Use 0 or 100 only if absolutely certain. Return ONLY strict JSON with EXACT keys (multi-box preferred): { 'label': 'yes'\|'no', 'confidence': integer 0–100, 'boxes': [[x1,y1,x2,y2], …] }. If 'label'='no', return an empty list for 'boxes'. If more than three candidates, return the best three. No prose, no code fences." | Defines the image coordinate system, the confidence rubric, and the JSON schema for the binary fracture decision and up to three bounding boxes. |

| **Seed** | **N patients** | **AUROC** | **Accuracy** | **MCC** | **Sensitivity** | **Specificity** |
| --- | --- | --- | --- | --- | --- | --- |
| 42 | 1,000 | 0.905 | 0.821 | 0.649 | 0.748 | 0.894 |
| 123 | 1,000 | 0.917 | 0.839 | 0.678 | 0.822 | 0.856 |
| 777 | 1,000 | 0.918 | 0.824 | 0.648 | 0.834 | 0.814 |
| 999 | 1,000 | 0.929 | 0.844 | 0.689 | 0.818 | 0.870 |
| 2024 | 1,000 | 0.924 | 0.837 | 0.676 | 0.878 | 0.796 |
| **Mean ± SD** | 1,000 | 0.9186 ± 0.0081 | 0.8330 ± 0.0089 | 0.668 ± 0.0165 | **0.820 ± 0.042** | **0.846 ± 0.036** |

**Supplementary Table S3. Robustness of the CNN baseline across five random seeds.**Patient-level metrics were computed on the identical balanced test set (N = 1,000) using the same Youden’s J operating threshold (t = 0.329) as in the main analysis. Metrics were consistent across runs (AUROC = 0.9186 ± 0.0081; Accuracy = 0.8330 ± 0.0089; MCC = 0.668 ± 0.0165; Sensitivity = 0.820 ± 0.042; Specificity = 0.846 ± 0.036; mean ± SD).

| Subgroup | N | CNN — Sens (95% CI) | GPT-4o — Sens (95% CI) | Claude — Sens (95% CI) | Gemini — Sens (95% CI) |
| --- | --- | --- | --- | --- | --- |
| All fracture-positive patients | 500 | 0.748 (0.709–0.786) | 0.854 (0.823–0.884) | 0.290 (0.252–0.330) | 0.402 (0.360–0.444) |
| AO severity /2 (predominantly buckle/torus) | 396 | 0.717 (0.672–0.763) | 0.836 (0.798–0.871) | 0.283 (0.240–0.326) | 0.376 (0.331–0.424) |
| AO severity /3 (displaced/complete) | 45 | 0.956 (0.889–1.000) | 0.911 (0.822–0.978) | 0.244 (0.133–0.378) | 0.556 (0.422–0.689) |
| AO severity /7 (Salter–Harris / epiphyseal) | 41 | 0.854 (0.732–0.951) | 0.780 (0.659–0.902) | 0.366 (0.220–0.512) | 0.415 (0.268–0.561) |
| AO severity /1 or /4 (other; small n) | 8 | 1.000 (1.000–1.000) | 0.875 (0.625–1.000) | 0.375 (0.125–0.750) | 0.500 (0.125–0.875) |
| Radius isolated (no ulna) | 362 | 0.704 (0.657–0.749) | 0.837 (0.801–0.876) | 0.279 (0.235–0.323) | 0.348 (0.298–0.398) |
| Any ulna involvement | 62 | 0.806 (0.710–0.903) | 0.806 (0.694–0.903) | 0.339 (0.226–0.452) | 0.532 (0.403–0.661) |

**Supplementary Table S4.** Patient-level sensitivity (95% bootstrap CIs, 2,000 resamples) by fracture subtype. AO severity '/2' = buckle/torus, '/3' = displaced/complete, '/7' = Salter–Harris/epiphyseal. CNN values are from the primary CNN run (seed 42).

| Model | Most common values (n) | Mean ± SD | Pred = yes (mean) | Pred = no (mean) | Correct (mean) | Incorrect (mean) | AUROC (95% CI) |
| --- | --- | --- | --- | --- | --- | --- | --- |
| GPT-4o | 90 (1313); 10 (751) | 60.9 ± 38.1 | 89.6 | 10.8 | 56.1 | 65.4 | 0.493 (0.482–0.505) |
| Claude 3.5 Sonnet | 95 (1146); 90 (384) | 79.1 ± 29.6 | 89.8 | 77.1 | 79.0 | 79.2 | 0.488 (0.465–0.511) |
| Gemini 1.5 Pro | 90 (2284); 95 (14) | 90.0 ± 0.4 | 90.1 | 90.0 | 90.0 | 90.0 | 0.518 (0.503–0.534) |

**Supplementary Table S5.** Confidence-score behavior of the three multimodal LLMs at the image level. Each LLM was queried separately for every radiograph and returned an independent confidence score. Signed confidence is defined as confidence/100 if the predicted label is "yes" and (100 − confidence)/100 otherwise. AUROC was computed against the image-level fracture ground truth and is reported with 95% confidence intervals from 2,000 patient-level cluster bootstrap resamples.

| Model | Pred. "yes" with box (n) | Mean IoU | Median IoU | Hit rate IoU ≥ 0.5 | Hit rate IoU ≥ 0.3 | Coverage IoU ≥ 0.5 (of 1,009) | Coverage IoU ≥ 0.3 (of 1,009) | Mean normalized center-distance |
| --- | --- | --- | --- | --- | --- | --- | --- | --- |
| GPT-4o | 636 | 0.072 | 0.041 | 2/636 (0.3%) | 10/636 (1.6%) | 0.2% | 1.0% | 0.072 |
| Claude 3.5 Sonnet | 132 | 0.022 | 0.000 | 0/132 (0.0%) | 2/132 (1.5%) | 0.0% | 0.2% | 0.144 |
| Gemini 1.5 Pro | 198 | 0.094 | 0.000 | 6/198 (3.0%) | 24/198 (12.1%) | 0.6% | 2.4% | 0.106 |

**Supplementary Table S6.** Quantitative localization performance of the three multimodal LLMs on fracture-positive images (n = 1,009). For images where the model predicted "yes" and returned at least one bounding box, the best-matching IoU and image-diagonal-normalized center-to-center distance to expert ground-truth annotations were computed. Hit rates are reported at IoU thresholds of 0.5 and 0.3. Coverage is the fraction of all 1,009 fracture-positive images for which the model produced a box satisfying the IoU threshold ≥ 0.3 and ≥ 0.5.
